# Diagnosis and Management of an H-type Tracheoesophageal Fistula in a Neonate: A Case Report

**DOI:** 10.7759/cureus.66618

**Published:** 2024-08-11

**Authors:** Ravi Reddy, Sai Bhavani Manchineni, Sagar Karotkar, Aditi Rawat, Chaitanya Kumar Javvaji

**Affiliations:** 1 Neonatology, Jawaharlal Nehru Medical College, Datta Meghe Institute of Higher Education and Research, Wardha, IND; 2 Pediatrics, Jawaharlal Nehru Medical College, Datta Meghe Institute of Higher Education and Research, Wardha, IND

**Keywords:** rigid and flexible video bronchoscopy, tracheoesophageal fistula, pediatric surgeon, contrast dye, tachypnea

## Abstract

Tracheoesophageal fistula (TEF) is a congenital anomaly resulting from the incomplete fusion of the tracheoesophageal ridge during the third week of embryonic development. This case report presents a male neonate, born at term via normal vaginal delivery, who developed respiratory distress, persistent cough, and vomiting within hours of birth. Despite initial management with respiratory support and antibiotics, the infant's condition persisted, prompting further investigation. High-resolution computed tomography and an esophagogram revealed a suspected H-type TEF, which was confirmed via rigid bronchoscopy. Following the diagnosis, the patient underwent corrective surgery, leading to symptom resolution. This case underscores the importance of considering TEF in neonates with persistent respiratory symptoms and the need for a combination of diagnostic modalities to confirm this rare anomaly. Prompt surgical intervention is crucial to prevent complications and improve outcomes.

## Introduction

An uneven trachea-esophagus connection is known as a tracheoesophageal fistula (TEF). A tracheoesophageal ridge fusion fails during the third week of embryonic development, establishing a TEF. Congenital TEF is an uncommon developmental abnormality [1]. The H subtype accounts for about 4% of TEFs [2]. Approximately one in 3500 live births is affected by it, and men are somewhat more likely to have it [3]. The "H-type" TEF is a rare and difficult-to-identify disorder. TEF is associated with esophageal atresia (EA) in 95% of instances. Its oblique route, which resembles an N, places it between the cranial orifice and the caudal orifice on the anterior wall of the esophagus and the posterior wall of the trachea [4]. The clinical presentation is characterized by three symptoms: paroxysmal coughing and cyanosis during feedings; recurrent chest infections and failure to thrive; and abdominal distention caused by gaseous loading of the intestines. If therapy is not administered, this syndrome may lead to other complications, such as chronic lung illness, failure to thrive, and recurrent pneumonia.

## Case presentation

**Figure 1:**
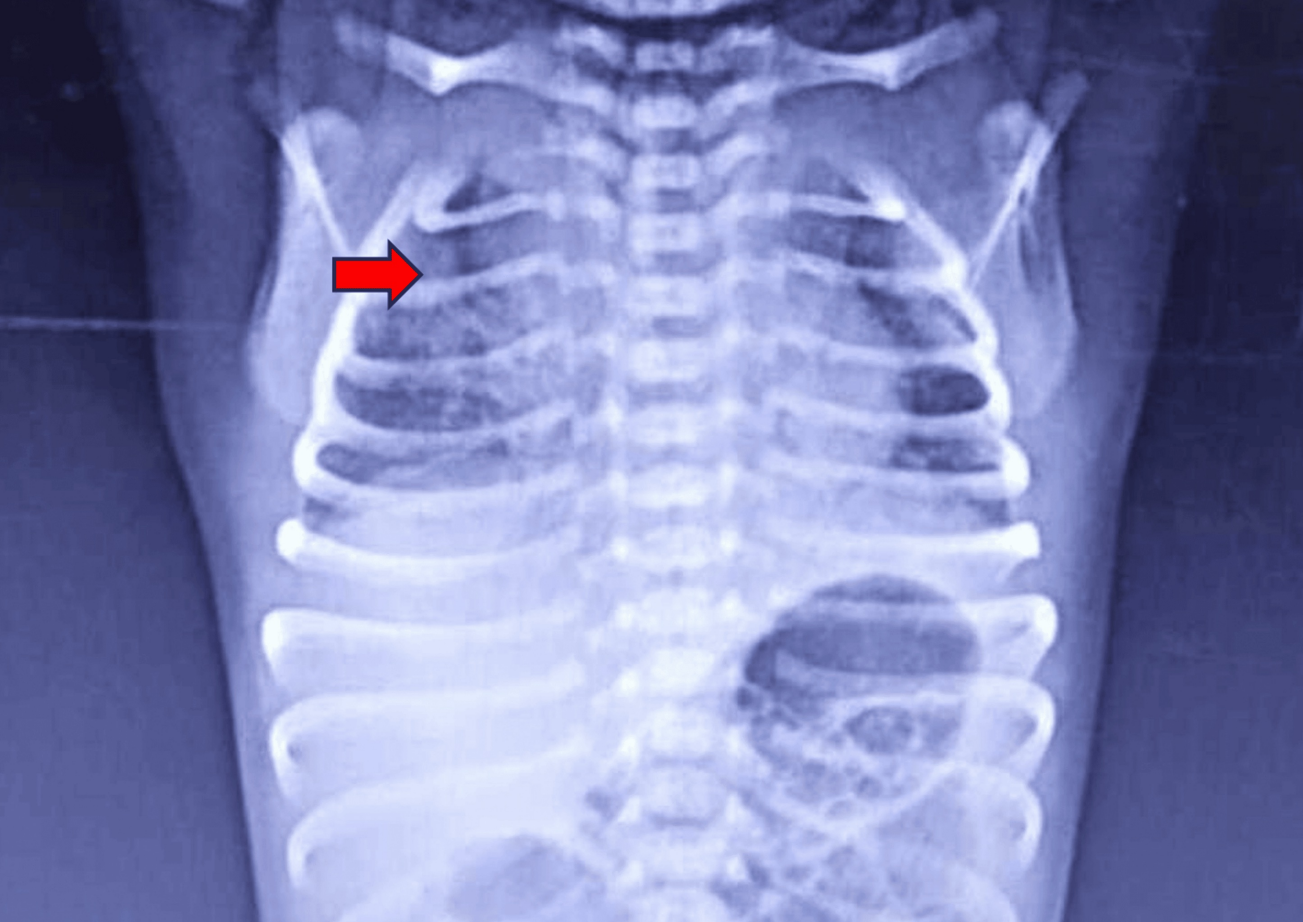
Chest X-ray showing homogenous opacities on the right side of the lung (red arrow)

A 3.2 kg male baby was born via normal vaginal delivery at term gestation; the baby cried immediately after birth and was shifted to the mother's side. At 16 hours of life, the baby had tachypnea and bilateral intercoastal and subcoastal retractions, along with a persistent cough and multiple episodes of vomiting. The baby was later shifted to the neonatal intensive care unit (NICU) at a local private hospital. The baby required respiratory support via nasal prongs. After preliminary investigations, the chest X-ray showed an air bronchogram and homogenous opacities on the right side (Figure 1). Because the baby's respiratory distress persisted and his saturation was not being maintained, the infant had to be intubated and placed on mechanical ventilation for two days. In addition to injection amikacin (15 mg/kg/dose) and syrup azithromycin (10 mg/kg/dose), the patient was started on injection piperacillin and tazobactam (100 mg/kg/dose) for four days. After some time, the patient's distress subsided, and the baby was extubated.

Later, the patient's tachypnea persisted. The baby had a cough and copious secretions. Antibiotics were escalated to meropenem (40 mg/kg/dose) throughout the nine-day hospital stay. On day 13, the baby was shifted to the NICU of our hospital for the above complaints. On respiratory system examination, the baby had tachypnea, copious secretions, and persistent cough, and bilateral coarse crepitations were present. Other system examinations were normal. The baby was taken on a heated humidified high-flow nasal cannula and started on injection piperacillin and tazobactam (100 mg/kg/dose) along with vancomycin (10 mg/kg/dose) added along with nebulization with injection adrenaline and N-acetylcysteine. Chest physiotherapy and neurophysiotherapy were initiated. High-resolution computed tomography (HRCT) was done, which is suggestive of ground-glass opacity and interlobular septal thickening in the right lower lobe.

**Figure 2:**
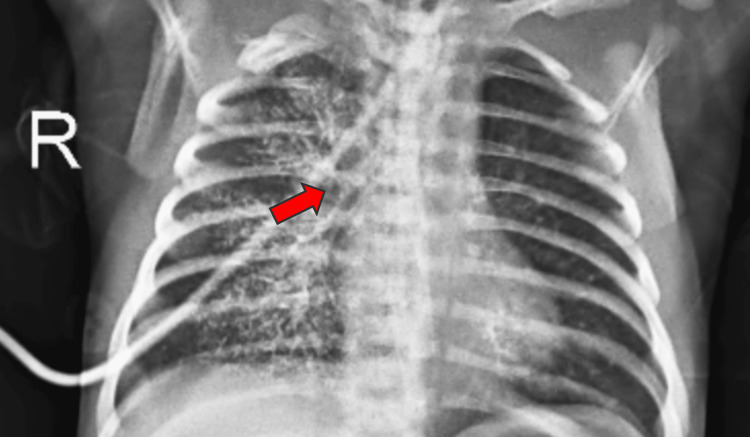
Chest X-ray taken one minute after giving contrast, showing the presence of contrast material in the right lung (red arrow)

**Figure 3:**
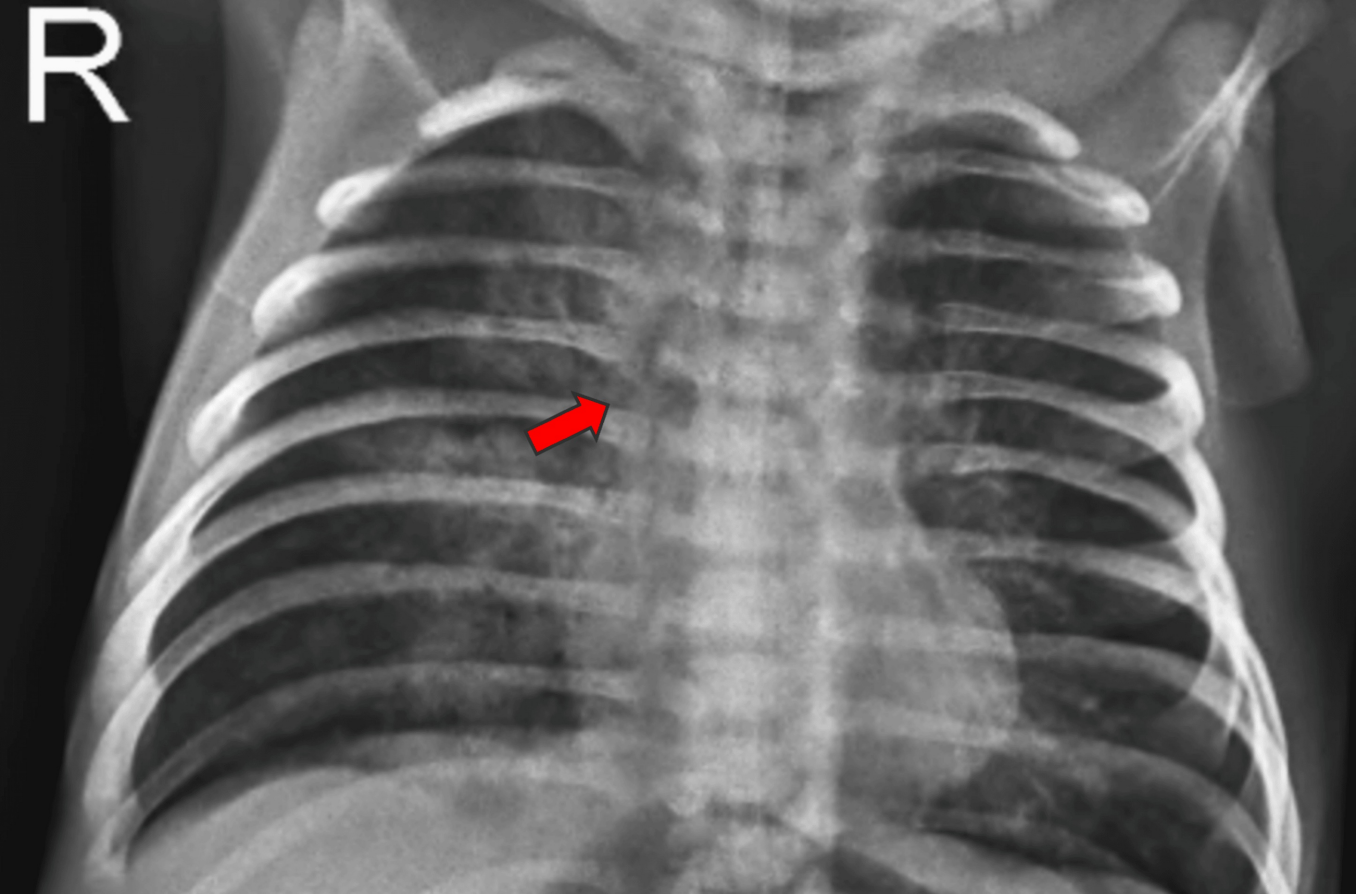
Chest X-ray taken 10 minutes after giving contrast showing the clearance of contrast (red arrow)

Chest X-ray showed a patchy consolidation with surrounding ground-glass opacities and interlobular septal thickening in the left lower lobe, suggesting an infective etiology. The patient developed repeated episodes of cough, tachypnea, and respiratory distress when oral feeds were initiated instead of nasogastric feeds. An esophagogram was done, which showed spillage of dye into the right lung suggestive of an H-type TEF. A contrast study was showing a contrast in the right lung suggestive of a fistula between the trachea and esophagus (Figure 2 and Figure 3).

Rigid bronchoscopy was done, which is suggestive of an H-type TEF. After the initial diagnosis, the patient was referred to pediatric surgery and was operated for the same, and later the symptoms were resolved post-corrective surgery. The baby is vitally and hemodynamically stable and was therefore discharged from the hospital with advice to follow up after 10 days.

## Discussion

An H-type TEF without EA is a rare entity seen in one in 50,000-80,000 live births and accounts for 4-5% of all congenital esophageal anomalies. Patients with this rare condition may not always be diagnosed in the immediate newborn period. Fistula anatomical diversity is the cause of delayed or missing diagnosis. No single study has the sensitivity and specificity needed to support an accurate "H-type" diagnosis. When an orogastric or nasogastric catheter cannot be inserted more than 10-15 cm into the stomach, the diagnosis of EA can be made. An anterior-posterior chest radiograph showing the catheter curled in the upper esophageal pouch can support this observation. A lateral chest radiograph will show a distal TEF; both views will show a gas-filled gastrointestinal tract.

A contrast esophagogram can help in diagnosis. The diagnosis of TEF can also be made using three-dimensional computed tomography scanning. To check for TEF, water-soluble contrast can be injected into the esophagus pouch while being guided by a fluoroscopic image. Avoid using barium contrast as it can cause aspiration pneumonia. It is imperative to remove the contrast material right away to prevent aspiration and regurgitation. Thickened water-soluble contrast material should be used in an upper gastrointestinal series to attempt diagnosing isolated TEF. The catheter is moved in a cephalad orientation after the distal esophagus has been filled. To find the TEF, esophageal endoscopy, bronchoscopy, and contrast swallow radiography should be employed [5]. It is possible to inject methylene blue into the trachea, which will cause a fistula to manifest in the esophagus.

A flawed division of the digestive and respiratory systems during embryogenesis leads to congenital TEF [6]. There are five anatomical types; the most prevalent one (90%) is proximal EA with distal TEF, and H-type TEF represents 4% of all tracheoesophageal abnormalities. The nonspecific presentation may cause a delay in the diagnosis. Investigations should be conducted to confirm the diagnosis and locate the fistula if there is a clinical suspicion of TEF. Endoscopic and radiological methods are required to verify the diagnosis. The nonspecific symptoms of H-type TEF and its rarity frequently lead to a delay in diagnosis [7,8].

Patients with a TEF frequently have concomitant malformations such as ventricular septal defect, duodenal atresia, and vertebral defects, anal atresia, cardiac defects, TEF, renal anomalies, and limb abnormalities (VACTERL). Isolated or H-type variants are uncommon. VACTERL defects may be ruled out via a renal ultrasound, spinal ultrasound, and limb radiography. Anastomotic leak, chronic second upper pouch fistula, esophageal stricture, recurrent fistula, persistent laryngeal nerve injury leading to voice cord paralysis, and mortality are among the complications after primary repair [9]. It is quite uncommon for a recurrent TEF to spontaneously close [10].

When a patient exhibits coughing fits that worsen during feedings and other symptoms including cyanosis, distended abdomen, choking episodes, recurrent respiratory infections, and underweight, a high index of suspicion is required for a diagnosis. H-type TEF must have been the cause of the coughing during oral feeding, but the tube feeding made the coughing better and covered up the telltale signs of TEF.

In addition to being diagnostic, tracheoesophageal bronchoscopy can help pinpoint the exact location of the fistula. Techniques for administering contrast and certain positions have been reported to help in diagnosis. The child should be in the prone position, and the test should be conducted with a nasogastric tube in situ to increase sensitivity. It is important to progressively remove the nasogastric tube when administering contrast. Aspiration pneumonia is a concern associated with contrast-enhanced investigations; thus, it is important to have enough emergency resuscitation equipment on available. The location of the fistula, the existence of a twin fistula, and the placement of the aortic arch can all be ascertained by bronchoscopy.

## Conclusions

This case study sheds light on the difficulties in diagnosing and the clinical complexity involved in the treatment of a neonatal H-type TEF. H-type TEF is uncommon and its presentation is rare in the neonatal age group. It warrants an increased clinical concern for babies who develop feeding difficulties, persistent coughing, and respiratory distress. It is important to identify small clinical signs and symptoms as soon as possible in order to diagnose it early with the correct diagnostic tests. Diagnostic methods such as bronchoscopy, contrast swallow tests, and HRCT are critical for both establishing the diagnosis and identifying the exact anatomical location of the fistula and its type. However, delays in diagnosis are common because of the nonspecific nature of signs and symptoms and the occurrence of H-TEF. This tells the significance of a multidisciplinary approach involving pediatric surgery, imaging, and surgical intervention.
